# Single Cell RNA-Seq Analysis Identifies Differentially Expressed Genes of Treg Cell in Early Treatment-Naive Rheumatoid Arthritis By Arsenic Trioxide

**DOI:** 10.3389/fphar.2021.656124

**Published:** 2021-05-24

**Authors:** Chunling Li, Tianshu Chu, Zhiyi Zhang, Yue Zhang

**Affiliations:** ^1^ Department of Rheumatology and Immunology, Henan Provincial People’s Hospital, People’s Hospital of Zhengzhou University, Zhengzhou, China; ^2^ Department of Rheumatology and Immunology, First Affiliated Hospital of Harbin Medical University, Harbin, China; ^3^ Department of Rheumatology, Immunology and Gerontology, South China Hospital, Health Science Center, Shenzhen University, Shenzhen, China; ^4^ Shenzhen Futian Hospital of Rheumatic Diseases, Shenzhen, China

**Keywords:** rheumatoid arthritis, arsenic trioxide, regulatory T cell, T helper 17 cell, single cell RNA-seq

## Abstract

**Objective:** Early treatment-naïve rheumatoid arthritis (RA) has defective regulatory T (Treg) cells and increased inflammation response. In this study, we aim to illustrate the regulation of Treg cells in pathogenesis of early rheumatoid arthritis by arsenic trioxide (As_2_O_3_).

**Methods:** We studied the effects of As_2_O_3_ on gene expression in early treatment-naïve RA Treg cells with single cell RNA-seq (scRNA-seq). Treg cells were sorted from peripheral blood mononuclear cells (PBMCs) and purified by fluorescence-activated cell sorting (FACS) and cultured with or without As_2_O_3_ (at 0.1 µM) for 24 h. Total RNA was isolated and sequenced, and functional analysis was performed against the Gene Ontology (GO) database. Results for selected genes were confirmed with RT-qPCR.

**Results:** As_2_O_3_ exerts no significant effect on CD4^+^ T-cell apoptosis under physical condition, and selectively modulate ^CD4+^ T cells toward Treg cells not Th17 cells under special polarizing stimulators. As_2_O_3_ increased the expression of 200 and reduced that of 272 genes with fold change (FC) 2.0 or greater. Several genes associated with inflammation, Treg-cell activation and differentiation as well as glucose and amino acids metabolism were among the most strongly affected genes. GO function analysis identified top ten ranked significant biological process (BPs), molecular functions (MFs), and cell components (CCs) in treatment and nontreatment Treg cells. In GO analysis, genes involved in the immunoregulation, cell apoptosis and cycle, inflammation, and cellular metabolism were enriched among the significantly affected genes. The KEGG pathway enrichment analysis identified the forkhead box O (FoxO) signal pathway, apoptosis, cytokine–cytokine receptor interaction, cell cycle, nuclear factor-kappa B (NF-κB) signaling pathway, tumor necrosis factor α (TNF-α), p53 signaling pathway, and phosphatidylinositol 3′-kinase (PI3K)-Akt signaling pathway were involved in the pathogenesis of early treatment-naïve RA.

**Conclusion:** This is the first study investigating the genome-wide effects of As_2_O_3_ on the gene expression of treatment-naïve Treg cells. In addition to clear anti-inflammatory and immunoregulation effects, As_2_O_3_ affect amino acids and glucose metabolism in Treg cells, an observation that might be particularly important in the metabolic phenotype of treatment-naïve RA.

## Introduction

Rheumatoid arthritis (RA) is a systemic autoimmune disease characterized by inflammatory synovitis and progressive destruction of joint cartilage and bone, leading to swelling, pain, stiffness, and loss of function (Han et al., 2008; Smolen et al., 2016). T cells are a critical regulator in the pathogenesis of RA as they accumulate in the lesions of joints, resulting in tissue-destruction and sustaining chronic inflammation (Goronzy and Weyand, 2017).

The pathological mechanism of RA remains unclear; however, it has been reported that the imbalance between T helper 17 (Th17) and regulatory T cells (Tregs) underlines the pathogenesis of RA, Th17 cells have proinflammatory effects, and the proportion of Th17 cells is higher in RA patients, and the content is positively correlated with the activity of RA disease (Niu et al., 2012; Jin et al., 2018; Li et al., 2019). Tregs, however, have immunosuppressive function and play an important role in the induction and maintenance of self-tolerance. The reduced content and dysfunction of Tregs are closely related to the occurrence and development of RA. Moreover, the connection of the Treg/Th17 cells imbalance is relevant for the development and/or progression of RA, which is in turn associated with the autoimmunity, chronic inflammation, and articular destruction in joints of RA patients (McInnes and Schett, 2007; Su et al., 2019). Previous literatures demonstrated that Treg cells mainly mediate the anti-inflammatory responses by producing IL-10 and transforming growth factor (TGF-β) suppression cytokines and maintain the state of autoimmune tolerance (Wang et al., 2017). IL-17 can induce proinflammatory cytokines production, chemokines (such as CCR6), and matrix metalloproteinases (such as MMP13), which result in tissue invasion and destruction as well as damage of articular cartilage and bone (Koenders et al., 2006; Miossec and Kolls, 2012). Tregs injected into collagen induced arthritis (CIA) mice can control the inflammatory responses and alleviate pathological damage (Safari et al., 2018). Thus, improving the Treg/Th17 cells balance shows some promise for the treatment of RA.

A couple of genome-wide association studies (GWAS) have previously been performed comparing RA either to osteoarthritis or to healthy donors (Stahl et al., 2010; Eyre et al., 2011). Two larger studies have utilized microarray and RNA-seq, respectively, to compare RA and healthy individuals CD4^+^ T cells, identifying a number of differentially expressed genes involved in differentiation, immune response, apoptosis, cell cycle regulation, and cellular metabolism (Sumitomo et al., 2018). Recent research has yielded biological therapies and small molecules to target signaling pathways and pathogenic components involved in inflammation and immunity, but in spite of these reasonably successful treatments, very few RA patients are able to achieve and stay in a state of drug-free remission. Innovative strategies are needed to obtain new insights into mechanisms which underline disease pathogenesis and to identify new treatments.

In current studies, As_2_O_3_ has shown substantial efficacy in treatment of patients with newly diagnosed or relapsed acute promyelocytic leukemia (APL) and other type of cancer and those characterized by the proliferation of immature immune cells, due to its substantial ability to induce apoptosis and mitogen activated protein kinase (MAPK) expression (Zhang et al., 2001). Furthermore, As_2_O_3_ exerts therapeutic effects on lymphoproliferative and severe autoimmune disease manifested in MRL/lpr mice (Bobé et al., 2006). Additionally, our previous accumulating data revealed that As_2_O_3_ contributes the balance of Treg/Th17 cells and affects both of their related signal cytokines pathways in treatment-naïve RA and CIA (Li et al., 2019a; Li et al., 2019b). Furthermore, we have discovered that As_2_O_3_ significantly suppress angiogenesis and induced fibroblast like synoviocytes (FLS) apoptosis in CIA and RA (Mei et al., 2011; Zhang et al., 2017). Moreover, our previous experiments found that A_2_O_3_ with vitamin D rescues the defective VDR-PPAR-γ functional module of autophagy synergistically in RA (Wang et al., 2019).

RA patients have an increased susceptibility to metabolic syndrome during the progression of their disease, associated with disruption of lipid and glucose metabolism (Cojocaru et al., 2012). There is also evidence on metabolic derangements in RA, and impairments in, for example, glycolysis, amino acids metabolism, oxidative stress, and mitochondrial respiration have been reported (Weyand and Goronzy, 2017; Falconer et al., 2018). Glycolysis deprivation was found to impair Th17 differentiation dramatically, while defective glycolysis supported the development of Treg cells. Replacement of glucose with galactose, treatment with 2-DG (an inhibitor of hexokinase, the first rate-limiting enzyme of glycolysis), and lack of crucial regulators of T-cell glycolytic metabolism, all resulted in diminished Th17 development but enhanced Treg-cell differentiation (Shi et al., 2011; Kalim et al., 2018; Kono et al., 2018; Cluxton et al., 2019). Conversely, inhibition of fatty acid oxidation results in diminished differentiation to Th17 cells, but increased development of Tregs (Gualdoni et al., 2016). Due to their established effects on these metabolic pathways in other cell types, As_2_O_3_ could plausibly affect RA pathogenesis via affecting Treg metabolism.

In the present study, we set out to study the effects of the As_2_O_3_ on gene expression in treatment-naïve RA Treg cells. The aim was to identify significantly modulated pathways and/or functional categories of genes that might be important in the pathogenesis of RA.

## Materials and Methods

### Early Treatment-Naïve RA Patient Collection

Treatment-naïve RA patients were obtained from the first affiliated hospital of Harbin Medical University, Department of Rheumatology. Recruited RA patients adhered to the Helsinki Declaration. All the three early treatment-naïve RA patients were seropositive for rheumatoid factor (RF) and or/anti-citrullinated peptide antibodies (ACPA), fulfilled 2010 ACR/EULAR criteria for RA and had active disease with a DAS28 > 3.2. The three early treatment-naïve RA patients were collected for RNA-seq and six treatment-naïve RA patients were collected for validation by qRT-PCR (Table 1). All the RA patients fulfilled the ACR criteria, according to clinical and radiological imaging (Aletaha et al., 2010). All participants gave written informed consent according to the Declaration of Helsinki. Ethics approval for the study was obtained from Harbin Medical University Research and Ethics Committee, Henan Provincial People’s Hospital Research and Ethics Committee, Zhengzhou University Research and Ethics Committee.

**TABLE 1:** General information of early treatment-naïve RA patients used for scRNA-seq and qRT-PCR analysis.

Clinical and laboratory characteristics of treatment-naïve RA* for scRNA-seq and qRT-PCR		
	scRNA-seq	qRT-PCR
Age, y	43 ± 4.52	44 ± 7.81
Sex (male/female)	1/2	1/5
Morning stiffness	2 (67)	5 (83)
Serum rheumatoid factor	3 (100)	4 (67)
Serum anti-CCP	3 (100)	5 (83)
Bone erosions	1 (33)	2 (33)
DAS28, mean ± S.E.M (range)	5.5 ± 0.4 (4.2-8.5)	5.7 ± 0.5 (4.0-8.9)
Swollen joint count, mean ± S.E.M (range)	5.2 ± 1.8 (2-8)	5.5 ± 1.9 (2-16)
Tender joint count, mean ± S.E.M (range)	4.9 ± 1.7 (2-10)	12.6 ± 2.5 (2-24)
Disease duration (month), mean ± S.E.M (range)	5.2 ± 1.6 (1-12)	7.6 ± 2.3 (2-24)

* Patients were diagnosed as having rheumatoid arthritis (RA) if they met ≥4 of criteria listed. Except where indicated otherwise, values are the number (%) of patients. Anti-CCP = anti-cyclic citrullinated peptide; DAS28 = Disease Activity Score in 28 joints.

### Apoptosis Assay

Naïve CD4^+^ T cells were isolated from early treatment-naïve RA patients and cultured in the presence or absence of As_2_O_3_ with anti-CD3/CD28 activation and IL-2 exists. The detailed procedures as described in our previous study (Li et al., 2019).

### Flow Cytometry

For intracellular cytokine detection, cells were stimulated with the corresponding Cell Activation Cocktail (with Brefeldin A) (Biolegend, San Diego, CA) for 6 h. The detailed procedures described in our previous study (Li et al., 2019).

### Cytokine Quantification

The cytokines IL-17A and IL-10 (D1700 and D1000, respectively, purchased from R&D systems, Minneapolis, United States) and MMP13 (E-EL-H0134c, Elabscience Biotechnology, Wuhan, China) were determined using enzyme-linked immunosorbent assay (ELISA). The ELISA was performed according to the manufacturer’s instructions.

### Immunofluorescence Staining

Cultured cells were fixed in 4% paraformaldehyde, followed by penetrating and blocking serum for 1 h, Rabbit anti-STAT3 (ab68153, Abcam, Cambridge, MA, United States) and Rabbit anti-Foxp3 (BA 2032–1, Boster, Wuhan, China) were used as the primary antibody. Samples were washed three times and incubated with FITC secondary antibodies (PV6001, ZSGB-BIO, Beijing, China), and DAPI (Sigma) was used for staining nuclei. Images were captured using a microscope (Leica, Mannheim, Germany).

### Single Treg Cell Sorting

**FIGURE 1:**
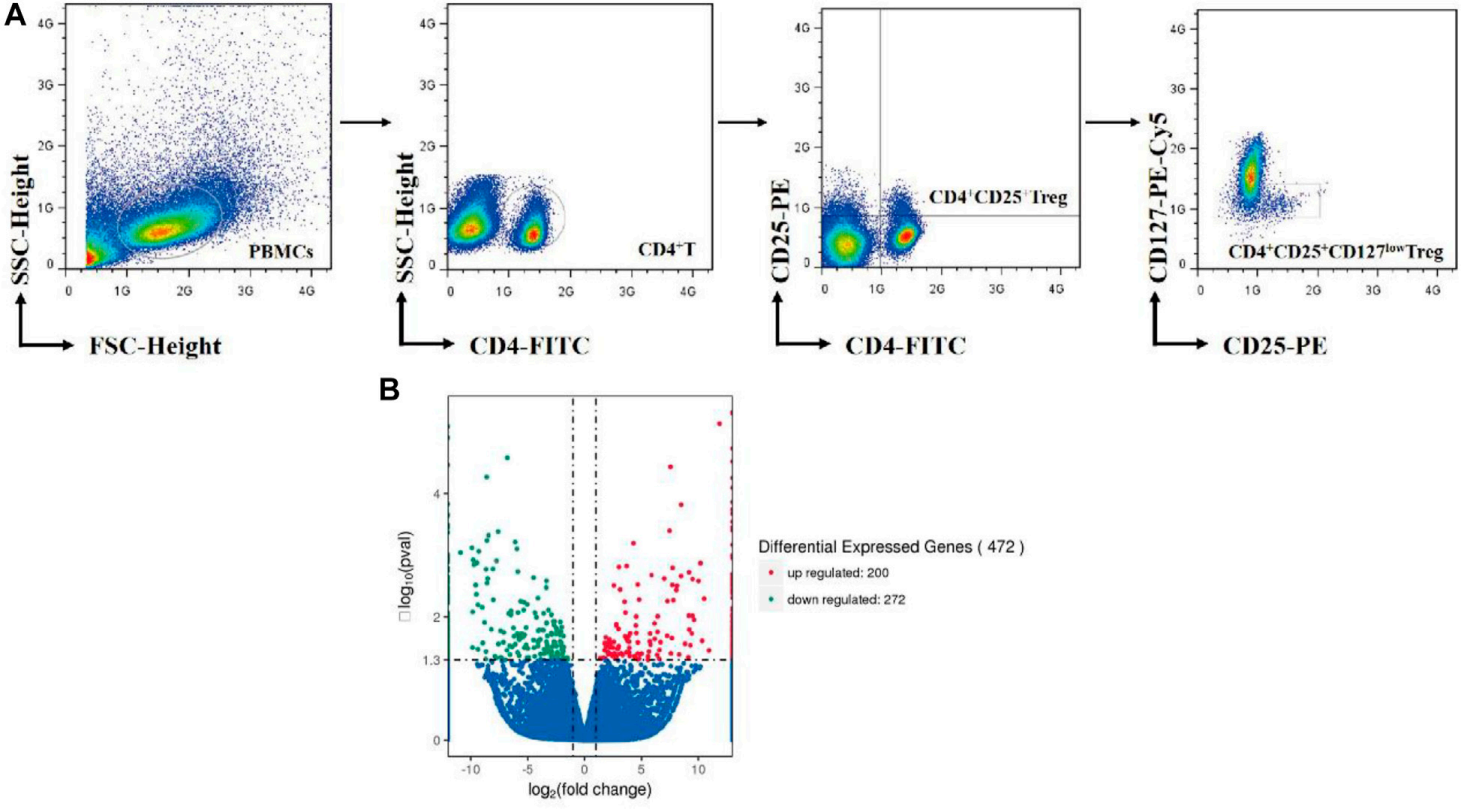
**(A)** Overview of Treg-cell isolation by cell sorter. **(B)** Volcano Plots include all differential expressed genes absolute value log2-fold change >0.5. Red nodes represent upregulation and blue nodes represent downregulation in treatment Treg cells compared to nontreatment Treg cells.

Treg cells were purified from PBMCs by high-speed cell sorter system (Moflo XDP, Beckman coulter, United States). The detailed procedures are described in our previous literature (Li et al., 2019). The working schematic model is shown in Figure 1A.

### RNA Quantification and Qualification

RNA degradation and contamination were monitored on 1% agarose gels. RNA purity was checked using the NanoPhotometer spectrophotometer (IMPLEN, CA, United States). RNA concentration was measured using Qubit RNA Assay Kit in Qubit 2.0 Flurometer (Life Technologies, CA, United States). RNA concentration and integrity were assessed using the RNA Nano 6000 Assay Kit of the Bioanalyzer 2100 system (Agilent Technologies, CA, United States).

### GO and KEGG Enrichment Analysis of Differentially Expressed Genes

To assess the function of identified DEGs, the functional analysis and clustering tool from Database for Annotation, Visualization and Integrated Discovery (DAVID) v6.7 was used to perform Gene Ontology (GO) function enriched analysis based on DEGs obtained from RA Treg cells. GO enrichment analysis of DEGs was implemented by the GOseq R package, in which gene length bias was corrected. DAVID provides a comprehensive set of functional annotation tools for investigators to understand the biological meaning based on a large list of genes (http://david.ncifcrf.gov/). For any given gene list, DAVID tools can identify enriched biological themes (GO terms), discover enriched functional-related genes cluster, and visualize genes on BioCarta and KEGG pathway maps (Huang et al., 2009). Therefore, DAVID was carried out to identify the enriched GO functions including the biological processes (BPs), molecular functions (MFs), and cell components (CCs). KEGG Orthology Based Annotation System (KOBAS 2.0) (http://kobas.cbi.edu.cn) was employed to identify biological pathways from the identified DEGs involved in the diseases. It performs statistical tests to identify statistically significantly enriched pathways and diseases using biological knowledge from five well-known pathway databases and GO (Xie et al., 2011). Thus, the KOBAS 2.0 was used to identify the enriched KEGG pathway based on adjusted *p* values. KEGG pathways including five or more DEGs genes were considered as the biologically meaningful analysis.

### Validating RNA-Seq Data Using Real-Time PCR

Total RNA was extracted from Treg cells according to the instructions of RNA extraction kit (Trizol Reagent, Invitrogen, Carlsbad, CA, United States). cDNA obtained from the reverse transcriptase reaction and subjected to quantitative RT-qPCR using SYBR Green PCR Master Mix (Bio-Rad, California, United States) and using the ABI Prism 7500 Sequence detection system (Applied Biosystems). Primers for human glyceraldehyde-3-phosphate dehydrogenase (GAPDH), cyclin-dependent kinase inhibitor 3(CDKN3), sushi domain containing 4 (SUSD4), histone cluster 4 H4 (HIST4H4), ubiquitin-specific peptidase 7 (USP7), histidine ammonia lyase (HAL), protein tyrosine phosphatase, non-receptor type 13 (PTPN13), DNA fragmentation factor subunit beta (DFFB), receptor interacting serine/threonine kinase 1 (RIPK1), and methyltransferase like 3 (METTL3) were purchased from Takara. The primer and concentrations were optimized according to the manufacturer’s instructions in SYBR Green PCR Master Mix Protocol. Relative expression levels of the nine selected genes were calculated by using the 2^−ΔΔCt^ method. The detailed following methods were performed according to our previous literature (Zhang et al., 2017).

### Statistical Analysis

Statistical significance was determined using GraphPad Prism Software (Version 6 for Windows; GraphPad Prism, San Diego, CA, United States). Simple comparisons were made using unpaired, two-tailed Student’s *t*-test for parametric data or Mann–Whitney test for nonparametric data, as indicated. Multigroup comparisons of the means were carried out by one-way analysis of variance test with post hoc contrasts by Tukey test. *p* values of 0.05 or less was considered statistically significant. All data are presented as mean ± S.E.M. Transcripts with significantly differential expressions of Treg cells by As_2_O_3_ were identified using hypergeometric test. The resulting *p*-values were adjusted using the Benjamini and Hochberg’s method for controlling the false discovery rate (FDR). DEGs were identified by applying the Benjamini and Hochberg method with adjusted *p* values of <0.05. DAVID v6.7 was used to carry out GO function enriched analysis based on the DEGs. KOBAS 2.0 was used to identify the enriched KEGG pathway based on the adjusted *p* values using Benjamini and Hochberg method.

## Results

### As_2_O_3_ Modulates IL-2 Production From PBMCs Without Affecting Viability

**FIGURE 2:**
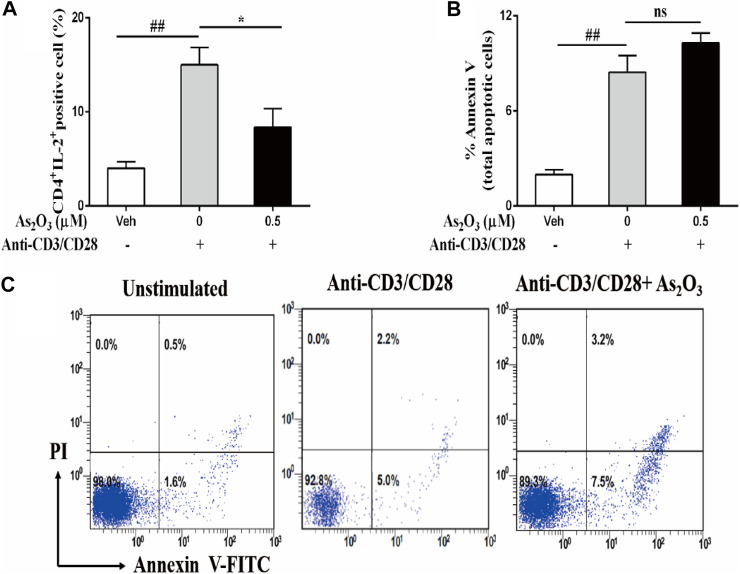
As_2_O_3_ suppress IL-2 expression from activated CD4^+^ T cells without affecting their viability. PBMCs (1 × 10^6^ cells per well) were left untreated or treated with As_2_O_3_ (0.5 µM) for 30 min. Cells were then stimulated with anti-CD3/CD28 for 8 h. Subsequently, cells were stained on the surface and intracellularly, and analyzed by flow cytometry. **(A)** Percentage of intracellular production of IL-2 from CD4^+^ T cell is shown as means ± SEM of six independent experiments. **(B,C)** Cell apoptosis of CD4^+^ T cells after activated with anti-CD3/CD28 through staining for annexin V and PI was detected by flow cytometry analysis. Data are means ± SEM of six independent experiments. ^#^ *p* < 0.05, ^##^ *p* < 0.01 *vs* nonactivated PBMCs cells; *p < 0.05, **p < 0.01 *vs* PBMCs nontreated with As_2_O_3_ (one-way ANOVA).

The immunomodulatory activity of As_2_O_3_ on PBMCs cells responses was also determined by the reduction of the growth factor IL-2 compared with IL-2-producing anti-CD3/CD28 activated PBMCs (Figure 2A). This effect was not due to an induction of the cell death, as assessed by annexin V staining that was used as a marker for apoptosis in combination with propidium iodide (PI), to distinguish between apoptotic and necrotic cells. After treatment 24 h, As_2_O_3_ treatment of PBMCs did not induce cell apoptosis, ruling out the potential cytotoxic role of As_2_O_3_ (Figures 2B,C). The decrease of IL-2 did not result in a significant decrease in PBMCs proliferation.

### As_2_O_3_ Critically Affect Th17 and Treg Differentiation and Related Signal Cytokines

**FIGURE 3:**
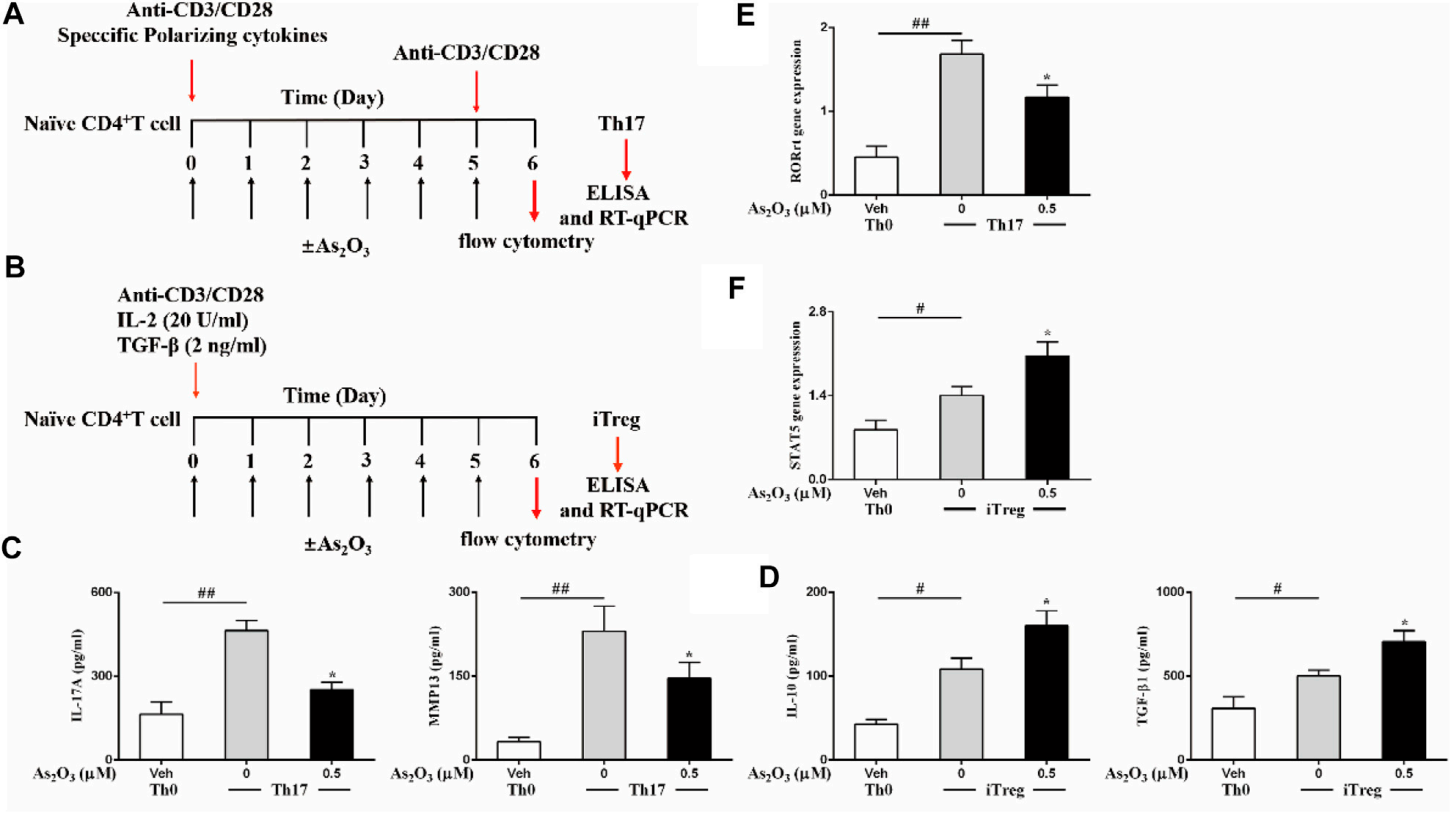
As_2_O_3_ affects Treg and Th17 cells differentiation. **(A,B)** Schematic representative of Th17 and Treg-cell differentiation. **(C,D)** ELISA of IL-17A and MMP13 in supernatants of Th17 cells polarized, IL-10 and TGF-β release in supernatants of Treg-cell production in the presence or absence of As_2_O_3_ (0.5 µM), measured after 24 h of restimulation with anti-CD3/CD28. **(E,F)** RT-qPCR analysis of the expression RORγt for Th17 cells, of STAT5 in Treg cells. Cycling threshold (CT) values are normalized to glyceraldehyde-3-phosphate dehydrogenase (GAPDH). Data are represented as means ± SEM of six independent experiments. ^#^ *p* < 0.05, ^##^ *p* < 0.01 vs. nonpolarized CD4^+^ T cells; *p < 0.05, **p < 0.01 vs. Th17 cells nontreated with As_2_O_3_ (one-way ANOVA).

Th17 and Treg subsets are both derived from naïve CD4^+^ T cells in peripheral blood upon antigen stimulation and specific polarizing cytokines. Because As_2_O_3_ dampened the inflammatory response of IL-17–producing cell from PBMCs, we next investigated whether As_2_O_3_ could directly affect their differentiation from naïve CD4^+^ T cells into Th17 cells lineages. To obtain this aim, a standard naïve CD4^+^ T cells differentiation assay was performed by polyclonal stimulation with anti-CD3/CD28 and specific polarizing cytokines in the presence of As_2_O_3_ (Figure 3A).

Under specific polarizing conditions, highly purified naïve CD4^+^ T cells displayed significantly higher amounts of intracellularly produced and extracellularly released IL-17 and MMP13, as compared to nonpolarized (Th0) cells (Figure 3C), in particular, in non-skewed Th0 cells, which produce low level IL-17 and MMP13. However, As_2_O_3_ significantly reduced Th17 generation, acting both on intracellular production and extracellular release from Th17 cells, suggesting that As_2_O_3_ affects not only Th17 cells induction but also specific functional properties. To address whether Th17 polarization was associated with the acquisition of their typical features, we also measured the mRNA encoding for the transcription factor RORγt known to be critical for their differentiation. As we expected, Th17 condition induced the highest expression of their specific transcription factors. The presence of As_2_O_3_ during Th17 polarization led to decreased RORγt in Th17 cells (Figure 3E). These findings support a pivotal role of As_2_O_3_ in hindering *de novo* Th17 differentiation.

In light of the role of As_2_O_3_ in resolving inflammation and because Treg cells is an important cell subset involved in modulating and maintaining self-regulation of the immune system, we also investigated whether As_2_O_3_ could affect the generation of induced Treg cells. This cell subset develops from naïve CD4^+^ T cells upon antigen stimulation and transforming growth factor-β (TGF-β) exposure. To obtain this aim, highly purified naïve CD4^+^ T cells were cultured under Treg-inducing conditions in the presence of As_2_O_3_ (Figure 3B). We found that As_2_O_3_ potentiated Treg differentiation, with As_2_O_3_ enhancing STAT5 expression compared to control Treg cells (Figure 3F). As_2_O_3_-induced *de novo* generation of Treg cells was also paralleled by their capacity to increase their suppressive cytokines IL-10 and TGF-β1 (Figure 3D), suggesting that As_2_O_3_ affect not only Treg induction but also specific functional properties.

### As_2_O_3_ Promotes CD4^+^CD25^+^ Treg Cells Proliferation

**FIGURE 4:**
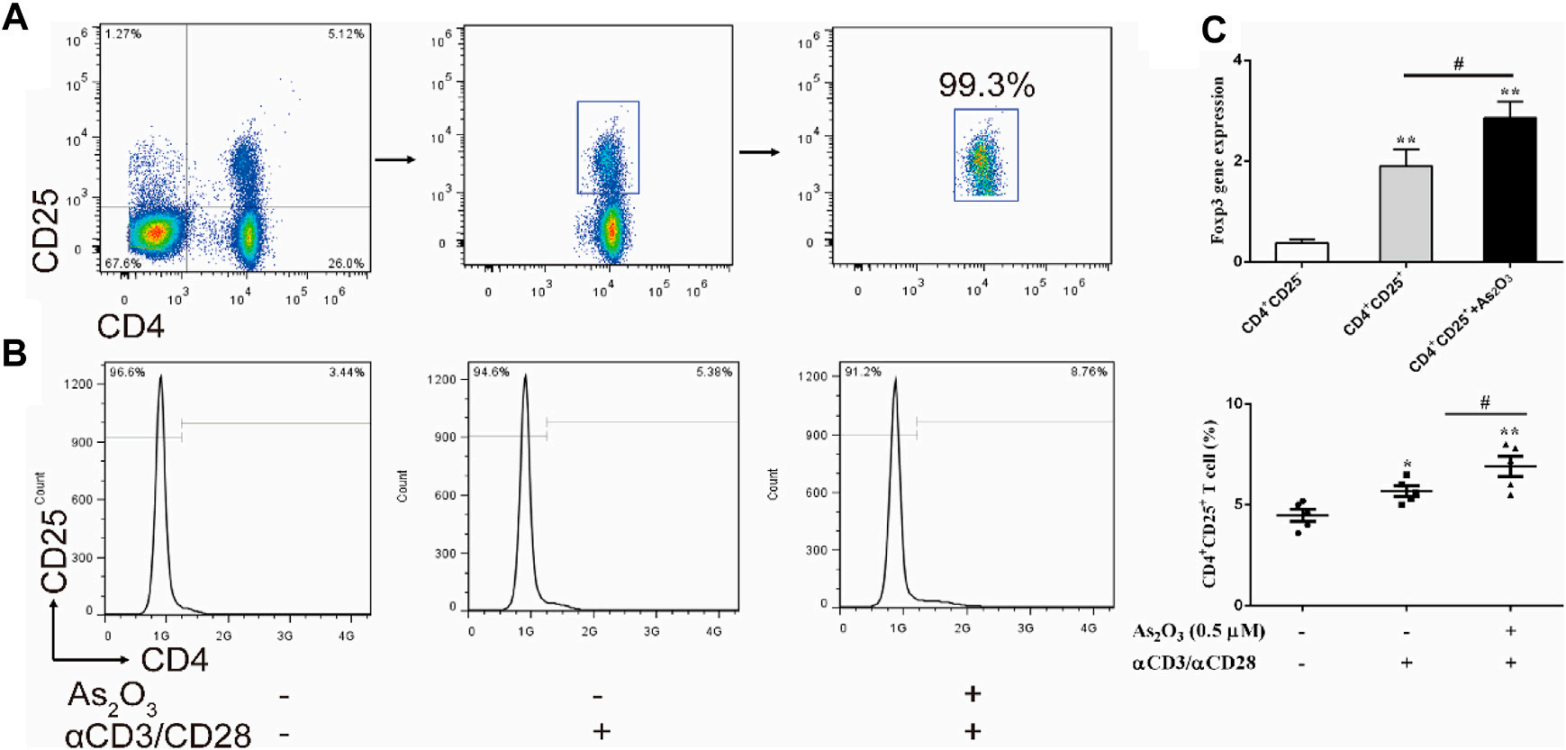
As_2_O_3_ increased CD4^+^CD25^+^ Treg-cell proportion by regulating Foxp3 expression. **(A)** CD4^+^CD25^+^ Treg cells and CD4^+^CD25^−^ T cells were isolated from PBMCs in treatment-naïve RA patients. The purity of obtained cells was ≥99% confirmed by flow cytometry. **(B)** Histograms represent the proportion of CD4^+^CD25^+^ Treg cells from treatment-naïve RA patients in absence or presence of As_2_O_3_ (0.5 μM) and with or without anti-CD3/CD28 activation *in vitro*. **(C)** qRT-PCR for detection of Foxp3 from purified CD4^+^CD25^+^ Treg-cell subsets. The statistical analysis was performed using one-way ANOVA. Data are represented as the mean ± SEM in six independent experiments. ^#^ *p* < 0.05, ^##^ *p* < 0.01 *vs* nontreatment cells; *p < 0.05, **p < 0.01 vs. CD4^+^CD25^−^ T cells from treatment-naïve RA patients.

To investigate the effect of As_2_O_3_ on CD4^+^CD25^+^ Treg cells proliferation, CD4^+^CD25^−^ T and CD4^+^CD25^+^ Treg cells were isolated from PBMCs in treatment-naïve RA patients (Figure 4A). CD4^+^CD25^+^ Treg cells generally represent anti-inflammatory subtype. To examine whether As_2_O_3_ directly affect CD4^+^CD25^+^ Treg-cell proliferation, CD4^+^CD25^+^ Treg cells were cultured with physical condition anti-CD3/CD28 stimulation, as well as As_2_O_3_ was added in the well for 3 days. Interestingly, we noticed that As_2_O_3_ dramatically increased the proportion of CD4^+^CD25^+^ Treg cells compared with without As_2_O_3_ treatment of CD4^+^CD25^+^ Treg cells (Figure 4B). To address whether As_2_O_3_ affects CD4^+^CD25^+^ Treg-cell polarization was associated with the acquisition of their typical features, we also measured the mRNA encoding for the transcription factor known to be critical for their differentiation Foxp3. As expected, As_2_O_3_ induced higher expression of Foxp3 compared with CD4^+^CD25^+^ Treg cells alone (Figure 4C).

### As_2_O_3_ Affects the Phenotype of Treg and Th17 Cells

**FIGURE 5:**
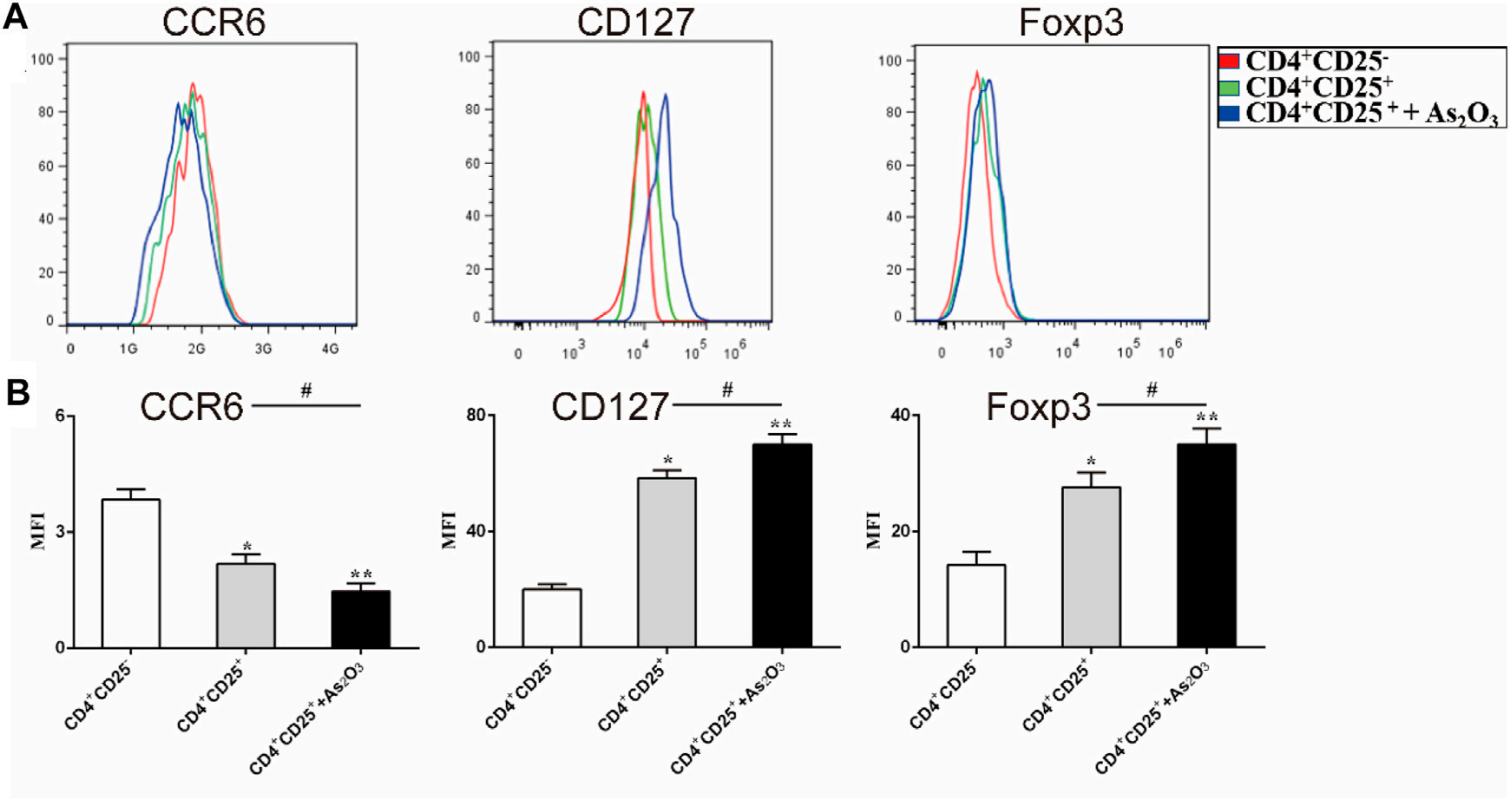
As_2_O_3_ affect phenotype of CD4^+^CD25^+^ Treg cells. **(A)** Histogram represent that surface expression of CCR6, CD127, and intracellular expression of Foxp3 were measured in CD4^+^CD25^−^ T cells, CD4^+^CD25^+^ Treg cells and coculture in the presence or absence of As_2_O_3_. **(C)** The expression levels of the surface and intracellular markers were quantified as mean fluorescence intensity (MFI). The statistical analysis was performed using one-way ANOVA. Data are represented as the mean ± SEM in six independent experiments. ^#^ *p* < 0.05, ^##^ *p* < 0.01 vs. nontreatment cells; *p < 0.05, **p < 0.01 vs. CD4^+^CD25^−^ T cells from treatment-naïve RA patients.

To examine the effect of As_2_O_3_ in the phenotypic characteristics of Treg cells, we used flow cytometry to assess the expression of Foxp3 and surface markers known to be associated with either Th17 or Treg cells. CCR6 is human Th17 cell marker. The expression of CCR6 was slightly but significantly lower in CD4^+^CD25^+^ Treg cells than in CD4^+^CD25^−^ Treg cells (mean fluorescence intensity (MFI): 2.18 ± 0.24 vs 3.84 ± 0.27, *p* < 0.05), whereas the expression levels of the Treg-associated regulatory molecule CD127 and Foxp3 were significantly higher in CD4^+^CD25^+^ Treg cells than in CD4^+^CD25^−^ Treg cells (MFI: 58.4 ± 2.65 vs 20.2 ± 1.7 and 22.2 ± 1.02 17.6 ± 2.5, respectively). Interestingly, we noticed that As_2_O_3_ downregulated CCR6 expression, while upregulated CD127 and Foxp3 expression in CD4^+^CD25^+^ Treg cells (Figures 5A,B). These results show that As_2_O_3_ enhances Treg-cell immunosuppression by regulating phenotype of Treg and Th17 cells.

### As_2_O_3_ Inhibited Nuclear Translocation of STAT3 in Early Treatment-Naïve RA Patients

**FIGURE 6:**
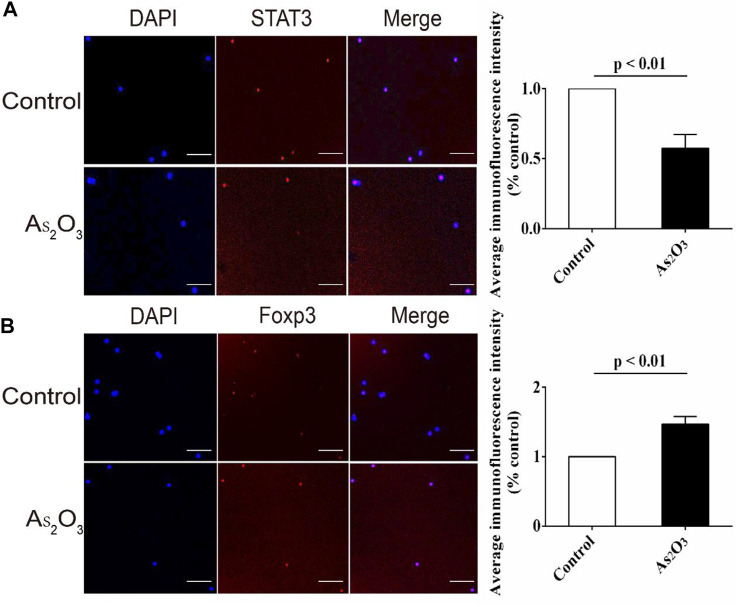
As_2_O_3_ increased gene expression of transcription factor Foxp3 for Treg cell while decreased STAT3 for Th17 cell from treatment-naïve RA patients. **(A,B)** PBMCs were extracted and cultured with anti-CD3/CD28 in presence or absence of As_2_O_3_ for 24 h. Representative images of anti-STAT3 and anti-Foxp3 fluorescence (red), nuclear DAPI (blue), and merged with the bright field (Merge) in vehicle and As_2_O_3_ treatment. Scale bars: 100 μm As_2_O_3_ decreased the apparent STAT3 fluorescence intensity while increased that of Foxp3 compared with vehicle group. STAT3 and Foxp3 levels were detected and quantified by the fluorescence microscopy. The statistical analysis was performed using one-way ANOVA. Data are represented as the mean ± SEM in six independent experiments.

We studied the effects of As_2_O_3_ on STAT3 in Th17 and Foxp3 in Treg cells by immunofluorescent staining. We determined the effects of As_2_O_3_ on STAT3 and Foxp3 function by observing nuclear translocation. As shown, STAT3 in nucleus was reduced by As_2_O_3_ treatment (Figure 6A), while the Foxp3 was increased (Figure 6B).

### Pathway Enrichment Analysis

The pathway enrichment analysis identified 22 differentially expressed pathways. Considering that the biological functions identified and previous study results for Treg cells, eight potential critical pathways participate in regulation of Treg-cell function by As_2_O_3_ from early treatment-naïve RA patients (Table 2). One upregulated enriched pathway found to be involved in FoxO acetylation which affects Foxp3 transcription and Treg-cell development. Seven downregulated enriched pathways were also determined, including apoptosis, cytokine–cytokine receptor interaction, cell cycle, PI3K-Akt signaling pathway, nuclear factor kappa-B signaling pathway, calcium signaling pathway, and p53 signaling pathway (Table 2).

**TABLE 2:** KEGG pathways enriched in treatment Treg cells of early treatment-naïve RA patients.

KEGG pathway	Class	Number of genes (%)	Nominal *p* value^a^	Adjusted *p* value^b^
Upregulated in treatment Treg group	—	—	—	—
FoxO signaling pathway	Signal transduction	26 (20.5)	9.5 × 10^−5^	1.4 × 10^−3^
Downregulated in treatment Treg group	—	—	—	—
Apoptosis	Development	46 (23.1)	3.4 × 10^−5^	4.4 × 10^−3^
Cell cycle	Development	35 (22.4)	5.3 × 10^−6^	5.9 × 10^−4^
Rheumatoid arthritis	Immune disease	28 (20.6)	5.4 × 10^−5^	7.3 × 10^−3^
Nuclear factor-kappa B signaling pathway	Signal transduction	24 (26.5)	1.8 × 10^−4^	3.5 × 10^−2^
Cytokine–cytokine receptor interaction	Immune system	27 (23.2)	1.6 × 10^−5^	7.7 × 10^−3^
T cell receptor signaling pathway	Signaling molecules and interaction	22(21.5)	5.7 × 10^−5^	6.7 × 10^−3^

a Nominal *p* value was calculated by hypergeometric test.

b Adjusted *p* values was corrected from nominal *p* values by Benjamini and Hochberg multiple testing correction.

### Differentially Expressed Genes

After normalization and correction for multiple testing, 100 genes were upregulated more than 2.0-fold in As_2_O_3_-treated cells compared to control cells, and 136 downregulated by the same factor (FC<-2.0). In total, 472 genes were found to be differentially expressed in As_2_O_3_-treated vs. control Treg cells in a statistically manner (FDR-corrected *p* value <0.05). We have identified 472 DEGs including 200 upregulated genes and 272 downregulated genes in treatment Treg cells compared to nontreatment Treg cells with As_2_O_3_ (Figure 1B). Twelve most strongly up- and downregulated genes are listed in Table 3.

**TABLE 3:** Eight most strongly up- and downregulated genes in As_2_O_3_-treated RA Treg cells relative to control.

Genes most strongly upregulated by As_2_O_3_				
Genes	Name	Function	Log_2_-fold change	Nominal *p* value^a^
CDKN3	Cyclin-dependent kinase inhibitor 3	Cell cycle	11.8	49 × 10^−2^
SUSD4	Sushi domain containing 4	Immunity	7.6	4.8 × 10^−2^
HIST4H4	Histone cluster 4 H4	Histone regulation	5.5	3.2 × 10^−2^
PSAT1	Phosphoserine aminotransferase 1	Amino acid synthesis	4.8	4.3 × 10^−2^
PDK-3	Pyruvate dehydrogenase kinase	Glucose and fatty acid metabolism	4.2	4.6 × 10^−2^
CRAT	Carnitine O-acetyltransferase	Fatty acid metabolism	3.7	3.8 × 10^−2^
USP7	Ubiquitin-specific peptidase 7	Ubiquitination metabolism	3.1	4.1 × 10^−2^
HAL	Histidine ammonia lyase	Histidine metabolism	2.5	1.8 × 10^−2^
**Genes most strongly downregulated by As_2_O_3_**				
Genes	Name	Function	Log_2_-fold change	Nominal *p* value^a^
DFFB	DNA fragmentation factor subunit beta	Regulate Apoptosis	−8.4	4.9 × 10^−2^
PTPN13	Protein tyrosine phosphatase, nonreceptor type 13	Regulate apoptosis	−7.6	2.5 × 10^−2^
RIPK1	Receptor interacting serine/threonine kinase 1	Regulate Apoptosis	−6.4	4.6 × 10^−2^
UBR5	Ubiquitin protein ligase E3 component n-recognin 5	DNA damage	−5.5	4.9 × 10^−2^
PBRM1	Polybromo 1	Negative cell proliferation	−5.2	3.3 × 10^−2^
METTL3	Methyltransferase like 3	RNA methylation	−4.1	6.1 × 10^−2^
SLC45A4	Solute carrier family 45 member 4	Anion transport	−3.3	4.9 × 10^−2^
FLCN	Folliculinum	GTPase activation	−2.4	4.7 × 10^−4^

a Nominal *p* value was calculated by Fisher’s exact test.

The list of the most strongly upregulated genes include genes involved in the regulation of cell proliferation, immunoregulation, apoptosis, and amino acids and glycolysis metabolism. Among the most strongly downregulated genes are those linked to cell proliferation and differentiation, extracellular signal activation, and inflammation.

### GO Function Enrichment Analysis

**FIGURE 7:**
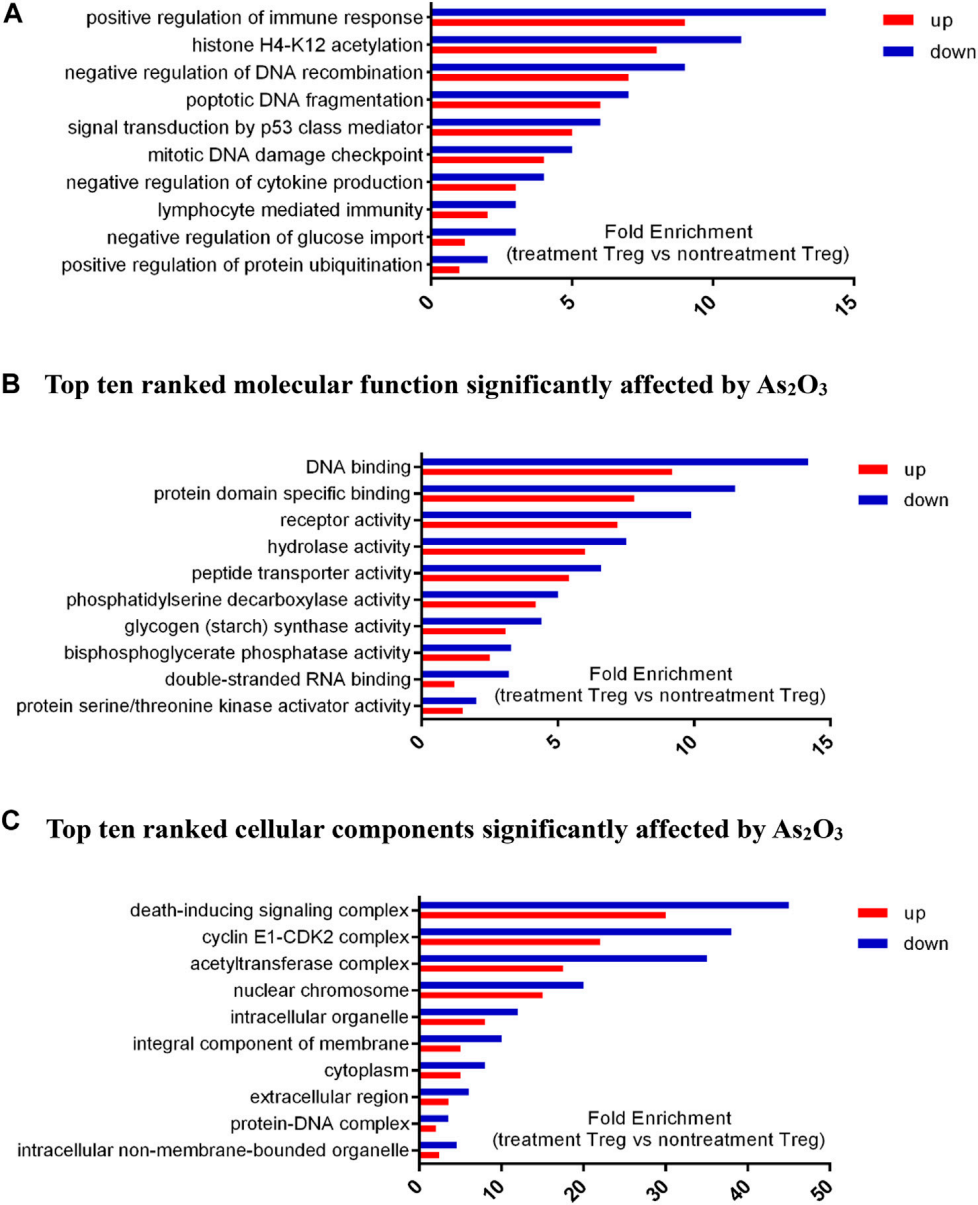
Differential expressed genes (DEGs) profiles of Gene Ontology (GO) terms (biological process, molecular function, and cellular component) in treatment Treg cells and nontreatment Treg cells from early treatment-naïve RA patients. **(A)** Top ten ranked biological process significantly affected by As_2_O_3._. **(B)** Top ten ranked molecular function significantly affected by As_2_O_3._. **(C)** Top ten ranked cellular components significantly affected by As_2_O_3_.

We selected nine significantly overrepresented BPs, including regulation of mitotic cell cycle, regulation of DNA recombination, and protein ubiquitination. There were nine significantly overrepresented MFs, including peptide transporter activity, RNA binding, glycogen synthase activity, kinase activator activity, and phosphatase activity. We also detected five significantly overrepresented CCs, including mitochondrial chromosome, cyclin E1–CDK2 complex, and DNA helicase complex (Figure 7). In summary, the significantly overrepresented BPs, MFs, and CCs were dramatically different between nontreatment Treg and treatment Treg cell with As_2_O_3_.

### Genes Involved in Inflammation and Immunoregulation

As chronic inflammatory autoimmune disease and changes in Treg cell frequency and dysfunction are central features in the pathogenesis, we set out to separately study genes linked to these processes (Table 4). Several proinflammatory factors such as hypoxia inducible factor 1 alpha subunit (HIF1α, fold change −8.4), matrix metallopeptidase 3 (MMP3, fold change −3.2), C-C motif chemokine receptor 6 (CCR6, fold change −7.6), and SMAD family member 3 (SMAD3, fold change −4.8) were significantly downregulated by As_2_O_3_, while the anti-inflammatory peroxisome proliferator activated receptor gamma (PPARγ, fold change 6.4) and interleukin 10 (IL-10, fold change 2.6) were upregulated. Genes affecting immune response, such as forkhead box O1 (Foxo1, fold change 11.8) and cytotoxic T-lymphocyte associated protein 4 (CTLA-4, fold change 4.6), were similarly upregulated.

**TABLE 4:** Selected genes linked to inflammation and immunoregulation in As_2_O_3_-treated RA Treg cells relative to control.

Inflammation and immunoregulation			
Genes	Name	Log_2_-fold change	Nominal *p* value^a^
Foxo1	Forkhead box O1	11.8	4.9 × 10^−2^
Foxp3	Forkhead box P3	8.6	1.3 × 10^−2^
CD28	CD28 molecule	6.5	4.3 × 10^−2^
PPARγ	Peroxisome proliferator activated receptor gamma	6.4	2.3 × 10^−2^
CD25	Interleukin 2 receptor subunit alpha	5.8	2.8 × 10^−2^
CTLA4	Cytotoxic T-lymphocyte associated protein 4	4.6	4.8 × 10^−2^
RUNX3	Runt related transcription factor 3	4.3	6.3 × 10^−2^
STAT5	Signal transducer and activator of transcription 5	3.7	4.6 × 10^−2^
TLR8	Toll like receptor 8	2.8	2.5 × 10^−2^
IL-10	Interleukin 10	2.6	2.5 × 10^−2^
TGF-β	Transforming growth factor beta 1	2.5	2.2 × 10^−2^
IL-15	Interleukin 15	2.1	4.1 × 10^−2^
STAT3	Signal transducer and activator of transcription 3	−9.4	6.1 × 10^−2^
RORc	RAR-related orphan receptor C	−8.8	4.9 × 10^−2^
HIF1α	Hypoxia inducible factor 1 alpha subunit	−8.4	4.6 × 10^−2^
CCR6	C–C motif chemokine receptor 6	−7.6	3.1 × 10^−2^
CXCR3	C-X-C motif chemokine receptor 3	−6.5	4.9 × 10^−2^
SOCS1	Suppressor of cytokine signaling 1	−6.0	3.3 × 10^−2^
IFN-γ	Interferon gamma	−5.3	4.9 × 10^−2^
SMAD3	SMAD family member 3	−4.8	4.7 × 10^−2^
IL-6	Interleukin 6	−4.4	4.7 × 10^−4^
MMP3	Matrix metallopeptidase 3	−3.2	2.6 × 10^−3^
IL-17A	Interleukin 17A	−2.5	3.4 × 10^−2^

a Nominal *p* value was calculated by Fisher’s exact test.

### Amino Acid and Carbohydrate Metabolism

RA is also known to be associated with metabolic syndrome. We separately studied genes for proteins participating in the main pathways of amino acid and carbohydrate metabolism (amino acid biosynthesis, glycolysis) (Weyand and Goronzy, 2017; Falconer et al., 2018). As_2_O_3_ did not have a marked (fold change>2.0) effect on any of these genes, with the single exception upregulation of acyl-CoA thioesterase 4 (ACOT4) (fold change 2.9).

### qRT-PCR Validates Differential Expressed Genes

**FIGURE 8:**
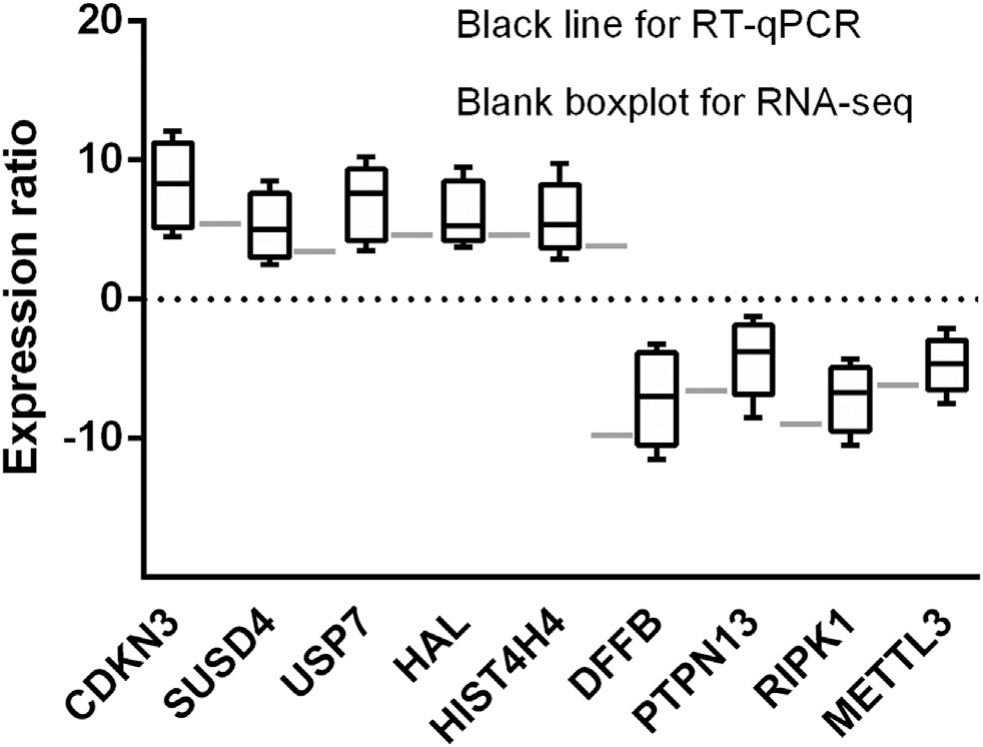
Histogram indicates the expression change of the nine selected genes detected by RT-qPCR (black bars, *n* = 6) and scRNA-seq (white bars, *n* = 3). The results showing the scRNA-seq analysis and RT-qPCR were consistent with expression change of the nine identified genes.

Five upregulated genes (CDKN3, SUSD4, USP7, HAL, and HIST4H4) and four downregulated gene (DFFB, PTPN13, RIPK1, and METTL3) in early treatment-naïve RA patients were selected for RT-qPCR. The nine selected genes expression were consistent between RNA-seq and RT-qPCR analysis, confirming the accuracy of our data (Figure 8).

## Discussion

In this study, we have presented a comprehensive analysis of the frequency, phenotype, cytokine profile, and gene expression profile of CD4^+^CD25^+^CD127^low^ Treg and Th17 cells from CD4^+^ T cells of PB from patients with early treatment-naïve RA patients.

Our findings have indicated that As_2_O_3_ increased CD4^+^CD25^+^ Treg-cell expression significantly in PBMCs of early treatment-naïve RA patients.

We report in this article for the first time, to our knowledge, that As_2_O_3_ can modulate CD4^+^CD25^+^ Treg-cell differentiation and affect its function, thus improving immune dysfunction. Audrey et al. reported that As_2_O_3_ increased the antitumor immune response through the depletion of Treg numbers mediated by oxidative stress in tumor-bearing mice (Thomas-Schoemann et al., 2012). Furthermore, our previous studies revealed that As_2_O_3_ could induce RA FLS apoptosis through NF-κB signaling pathway (Mei et al., 2011), inhibiting angiogenesis via modulating TSP-1-TGF-β-CTGF-VEGF functional module (Zhang et al., 2017) and rescuing the defective VDR-PPARγ functional module of autophagy (Wang et al., 2019). We also systematically reviewed that As_2_O_3_ is a feasible treatment option based on its ability to protect against inflammation (Zhang et al., 2018). Therefore, it is becoming increasingly clear that As_2_O_3_ might take part in the control of immune response by promoting the differentiation of CD4^+^CD25^+^ Treg cells and related phenotype expression.

Based on our previous findings, we conducted scRNA-seq analysis elicited the exact therapeutic mechanism of Treg cells by As_2_O_3_ in early treatment-naïve RA patients. As_2_O_3_ was found to affect the expression of a large number of genes in early treatment-naïve RA Treg cells. Among the most strongly affected genes were several involved in inflammation, immunoregulation, and amino acid and carbohydrate metabolism.

Immune imbalance along with autoimmune dysfunction is a central feature of RA. In our data, As_2_O_3_ reduced the expression of well-known Th17 transcription factor such as signal transducer and activator of transcription 3 (STAT3). In addition, As_2_O_3_ downregulated the expression of hypoxia-inducible factor 1 alpha subunit (HIF1α). As the relative expression of Treg/Th17–related cytokines during the course of RA, the effects of As_2_O_3_ on immune balance are likely to depend on the phase of the disease process.

RA has been reported to be associated with Treg/Th17 cell imbalance (Li et al., 2019a; Li et al., 2019b). Activation of the Foxo signaling pathway appears to counteract these effects and particularly, forkhead box O1 (Foxo1) has been shown to enhance Treg-cell differentiation and stability (Kerdiles et al., 2010). Interestingly, the expression of Foxo1 was strongly upregulated by As_2_O_3_ in the present data. Furthermore, As_2_O_3_ strongly enhanced the expression of Runx3, which has been shown to regulate Foxp3 expression (Loo et al., 2020).

These genes were predicted to target Foxo signaling pathway that play a major role in Treg-cell formation and function. Foxo induces the transcription factor Foxp3 expression. Single-cell RAN-seq revealed that the upregulated pathway mainly involves the Foxo signaling pathway–related protein acetylation and deubiquitination in Treg cell from early treatment-naïve RA patients by As_2_O_3_. The downregulated pathway mainly involves apoptosis activated by DNA fragmentation factor, cyclin E1, ATPase plasma membrane Ca2^+^ transporting 4, mitochondrial ribosomal protein, ubiquitin protein ligase in intracellular membrane, and extracellular region. Considering above of the findings, metabolic disorder and epigenetic modification may involve in the regulation of Treg cell by As_2_O_3_ from early treatment-naïve RA patients.

The highly conserved role of Foxo transcription factor in cell cycle inhibition and apoptosis has been extensively studied in the past decade. Previous literature has uncovered that Foxo transcription factors in T-cell fate specification, especially with regard to Treg-cell differentiation play a critical role by integrating PI3K-Akt and TGF-β-SMAD signaling pathways (Kerdiles et al., 2010). Preclinical studies demonstrated that dysfunctional differentiation of naïve T cell in RA patients is critically induced by insufficient of the histone acetyltransferase, which leads to the deficiency of Foxp3 acetylation and subsequently Foxp3 degradation (Su et al., 2019). Other researchers have previously shown that Foxp3 deubiquitination partially but significantly increase Treg cells and that partial rescue of Treg-cell development (Zhao et al., 2015). Therefore, we gain a hypothesis that As_2_O_3_ may affect Treg-cell function by enhancing Foxo acetylation and attenuating ubiquitination subsequently orchestrate a program of Foxp3 gene expression and Treg-cell differentiation. Thirteen identified MFs imply that As_2_O_3_ promotes Treg-cell differentiation and enhanced function may partially affect its epidemic modification. Foxo transcription factor genetic program controls aspects of Treg-cell differentiation and that are dispensable for the maintenance for Foxp3 expression and Foxp3^+^ cells expansion in response to homeostatic or inflammatory cues. Substrate-binding F-box protein SKP2 binds to ubiquitin ligase induces Foxo ubiquitination and subsequent proteasome degradation of Foxo (Huang and Tindall, 2011). This study revealed that As_2_O_3_ perhaps increase Treg differentiation and development by enhance Foxo acetylation and inhibit Foxo ubiquitination.

Our previous studies revealed that As_2_O_3_ at low concentration (0.1–0.5 µM) primarily promote Treg-cell differentiation and does not induce its apoptosis. RNA-seq analysis showed that As_2_O_3_ inhibits apoptosis which manifested tightly to the inactivation of cytokine–cytokine receptor interaction, cell cycle, PI3K-Akt signaling pathway, NK-κB signaling pathway, calcium signaling pathway, and p53 signaling pathway. Our data suggested that As_2_O_3_ perhaps attenuates DNA fragmentation factor subunit beta (DFFB) activity subsequently inhibit apoptosis (Han et al., 2020). Furthermore, As_2_O_3_ interaction may prevent apoptosis-inducing factor oxidation in mitochondria exposed to stress and undergoing apoptosis (Bano and Prehn, 2018). As_2_O_3_ tampered released NF-κB dimers activated through various posttranslational modifications and translocate to the nucleus where they bind to specific DNA sequences and inhibit transcription of target genes (Hayden and Ghosh, 2008).

An interesting example is pyruvate dehydrogenase kinase 1 (PDK1), which was one of the genes most strongly downregulated by As_2_O_3_. As this gene inactivates pyruvate dehydrogenase and prevents pyruvate convert into lactate or acetyl-CoA, inhibition of PDK1 during Th17-cell polarization by dichloroacetate (DCA) is sufficient to block Th17-cell differentiation (Bantug et al., 2018). Several genes promoting amino acid synthesis and transport, such as solute carrier family 1 member 5 (SLC1A5), which encodes a glutamine transporter and limits Th17-cell generation while promote Treg-cell generation (Nakaya et al., 2014), were also downregulated. However, genes coding for the enzymes participating in the major pathways of carbohydrate and amino acid metabolism (glycolysis and amino biosynthesis) were not significantly affected. Of the RA-associated glycolysis metabolism genes, glucose transporter (GLUT1) is a central mediator of carbohydrate metabolism. Its expression has been shown to be increased in RA (Falconer et al., 2018), and GLUT1 was found to be downregulated by As_2_O_3_ in the present study.

Cell cycle kinase, serine–protein kinase (ATM), is involved in the amino acid metabolism. T cells from RA that have low levels of ATM commit to the Th1 and Th17, rather than Treg (Weyand and Goronzy, 2017). These are examples of As_2_O_3_-induced normalization of the expression of carbohydrate and amino acid metabolism–related genes in RA Treg cells.

## Conclusion

In conclusion, As_2_O_3_ was found to cause a major phenotype switch in RA Treg. This is the first study investigating the genome-wide effects of As_2_O_3_ on the gene expression of treatment naïve Treg cells. In addition to promoting apoptosis, inhibiting angiogenesis, restoring immune imbalance, suppressing inflammation. As_2_O_3_ was also found to affect glucose-amino acid metabolism related genes. Since, targeting metabolism may be the potential therapeutic for treatment-naïve RA patients.

## Data Availability

The raw data supporting the conclusions of this article will be made available by the authors, without undue reservation.
