# Supplementary figures and images for: Single Cell RNA-Seq Analysis Identifies Differentially Expressed Genes of Treg Cell in Early Treatment-Naive Rheumatoid Arthritis By Arsenic Trioxide
Source: Front Pharmacol. 2021 May 24;12:656124. doi: 10.3389/fphar.2021.656124 (PMC8181733; doi:10.3389/fphar.2021.656124)

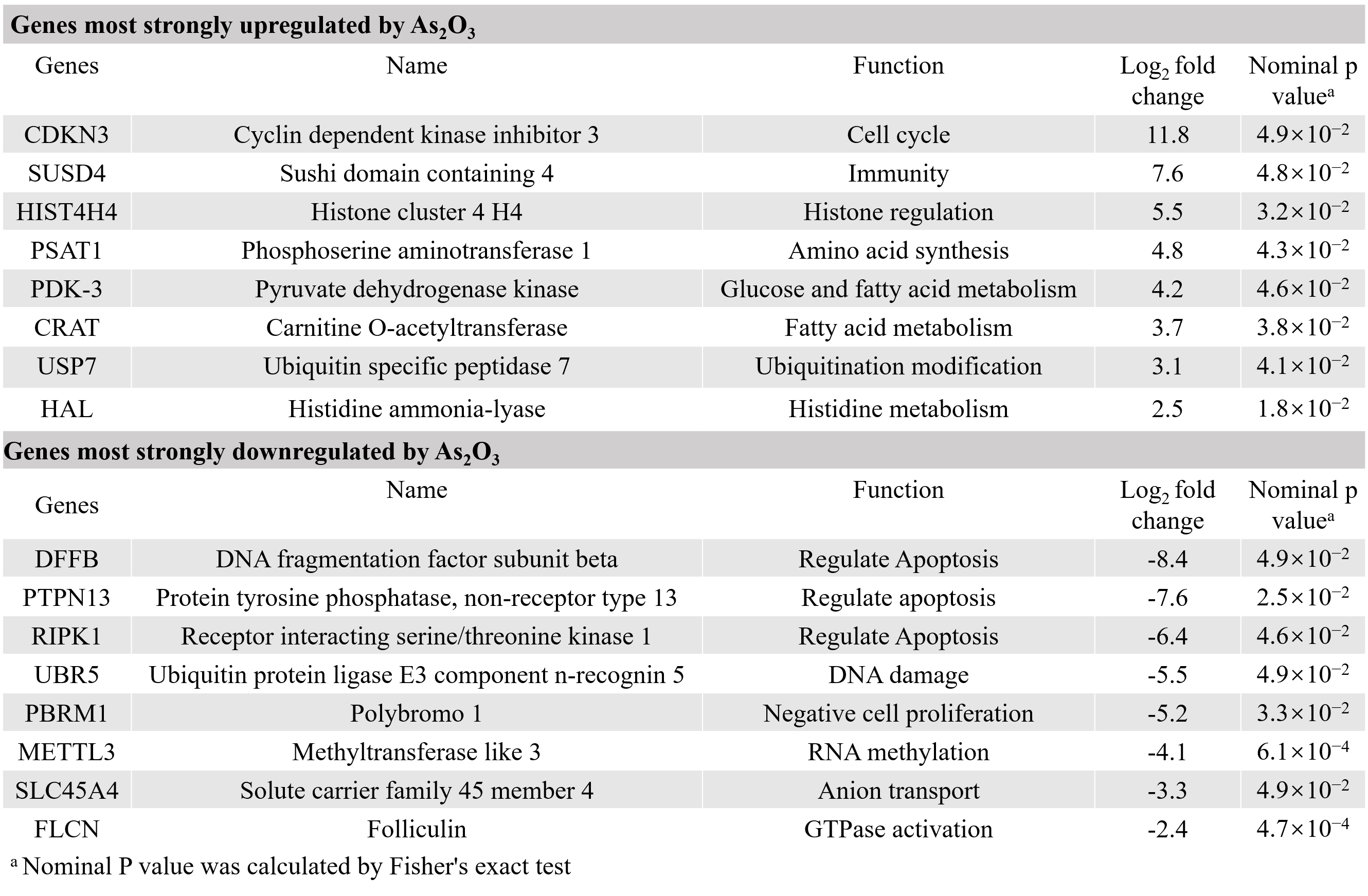

Supplement: Supplementary file 1 [file Image3.TIF]

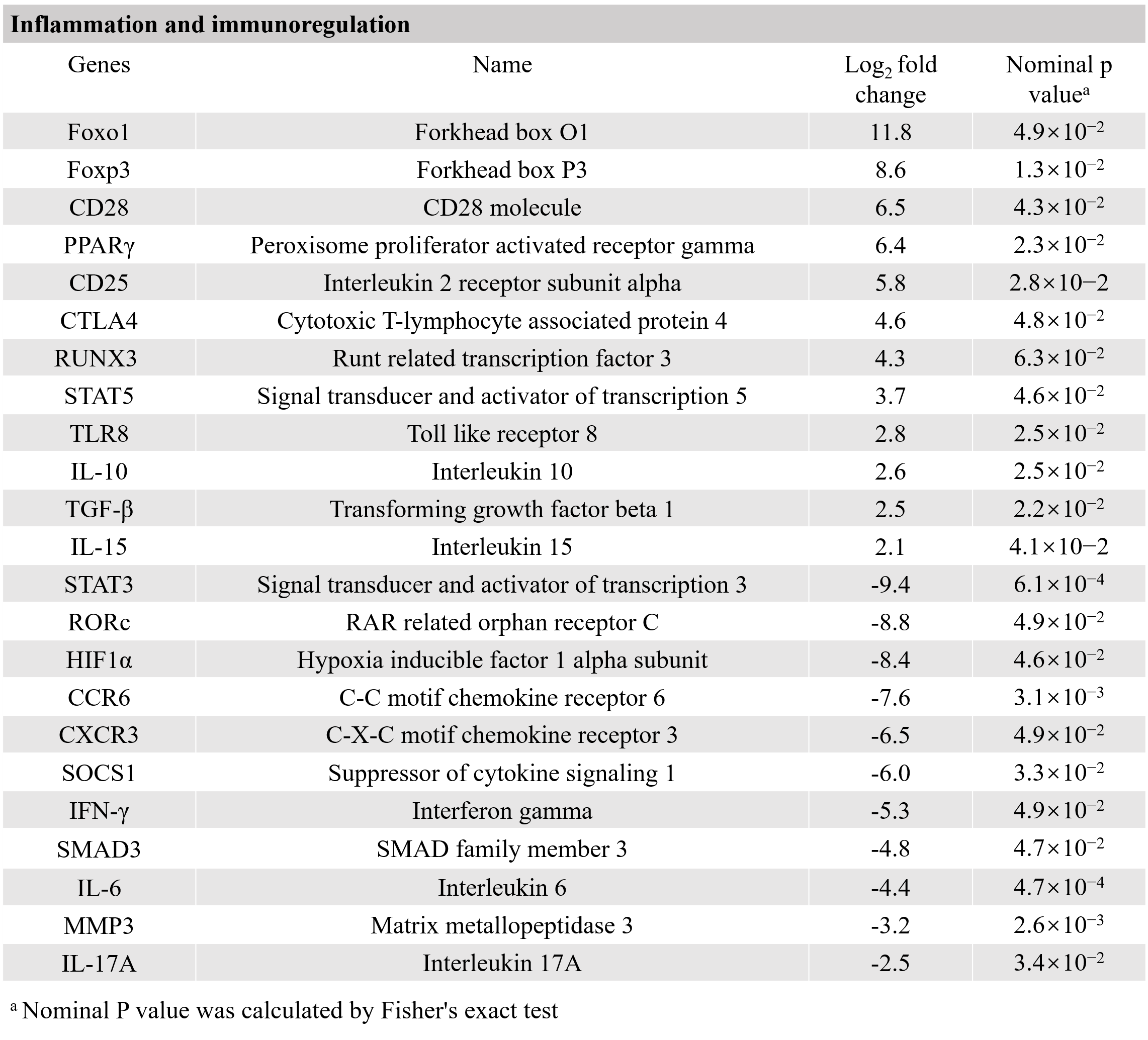

Supplement: Supplementary file 2 [file Image4.TIF]

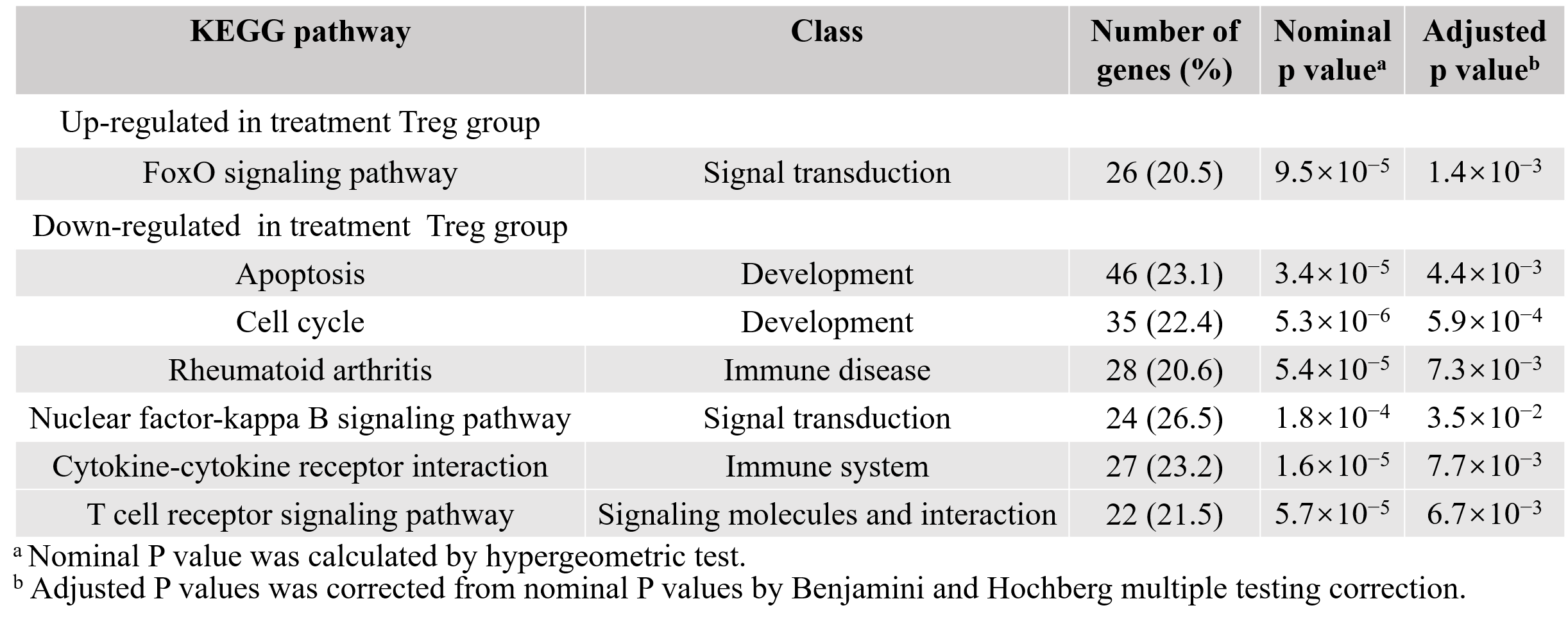

Supplement: Supplementary file 3 [file Image2.TIF]

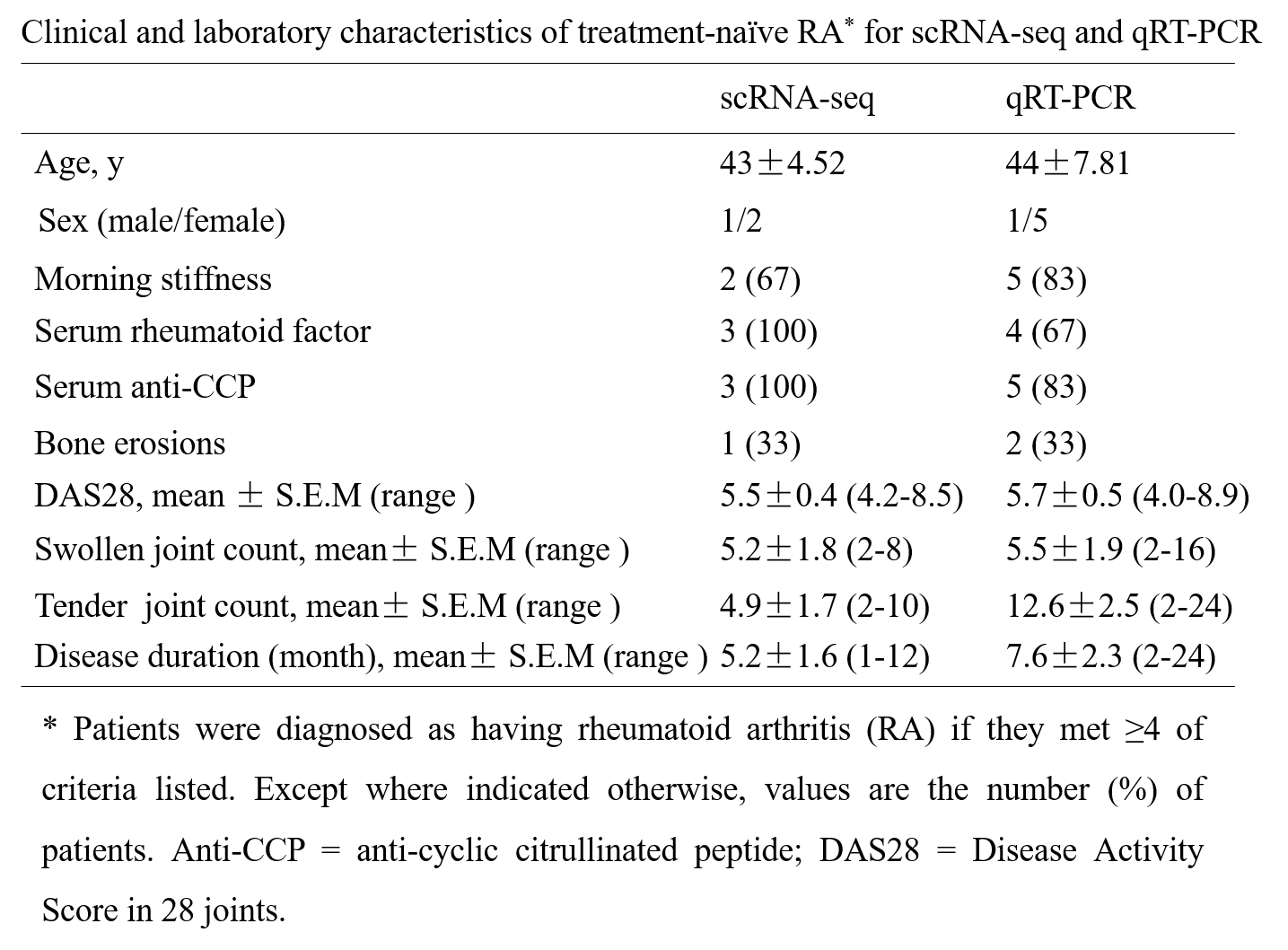

Supplement: Supplementary file 4 [file Image1.TIF]
